# NMR Studies of Aromatic Ring Flips to Probe Conformational Fluctuations in Proteins

**DOI:** 10.1021/acs.jpcb.2c07258

**Published:** 2023-01-14

**Authors:** Mikael Akke, Ulrich Weininger

**Affiliations:** † Division of Biophysical Chemistry, Center for Molecular Protein Science, Department of Chemistry, Lund University, P.O. Box 124, SE-221 00 Lund, Sweden; ‡ Institute of Physics, Biophysics, Martin-Luther-University Halle-Wittenberg, D-06129 Halle (Saale), Germany

## Abstract

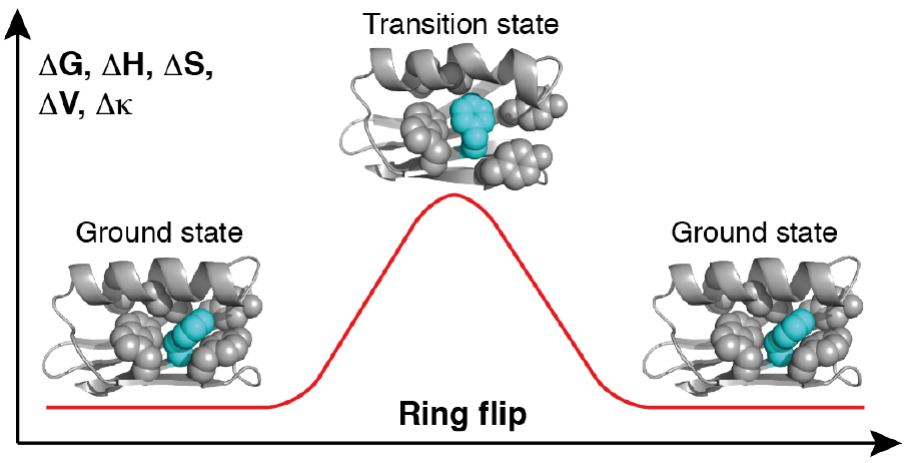

Aromatic residues form a significant part of the protein core, where they make tight interactions with multiple surrounding side chains. Despite the dense packing of internal side chains, the aromatic rings of phenylalanine and tyrosine residues undergo 180° rotations, or flips, which are mediated by transient and large-scale “breathing” motions that generate sufficient void volume around the aromatic ring. Forty years after the seminal work by Wagner and Wüthrich, NMR studies of aromatic ring flips are now undergoing a renaissance as a powerful means of probing fundamental dynamic properties of proteins. Recent developments of improved NMR methods and isotope labeling schemes have enabled a number of advances in addressing the mechanisms and energetics of aromatic ring flips. The nature of the transition states associated with ring flips can be described by thermodynamic activation parameters, including the activation enthalpy, activation entropy, activation volume, and also the isothermal volume compressibility of activation. Consequently, it is of great interest to study how ring flip rate constants and activation parameters might vary with protein structure and external conditions like temperature and pressure. The field is beginning to gather such data for aromatic residues in a variety of environments, ranging from surface exposed to buried. In the future, the combination of solution and solid-state NMR spectroscopy together with molecular dynamics simulations and other computational approaches is likely to provide detailed information about the coupled dynamics of aromatic rings and neighboring residues. In this Perspective, we highlight recent developments and provide an outlook toward the future.

## Introduction

Proteins are dynamic molecules that undergo conformational transitions often linked to biological function.^1−4^ Functionally important protein dynamics commonly require collective movement of many atoms that take place on relatively slow time scales, on the order of microseconds to milliseconds. Over the past two to three decades, advances in NMR methodology have made it possible to probe conformational fluctuations on such slower time scales via relaxation dispersion experiments designed for specific molecular moieties, such as the protein backbone or side chain methyl groups. Notably, these methods enable us to simultaneously characterize thermodynamic equilibria by determining the relative populations of alternative states, kinetics by determining rate constants of exchange between the states, and structure by determining differences in chemical shift or coupling constants between the states. NMR relaxation dispersion methods have revealed very important information about the functional role of minor, high-energy conformational states of proteins and nucleic acids.^4−8^ The thermodynamic, kinetic, and structural data can be complemented by detailed mechanistic insights from molecular dynamics (MD) simulations that can be validated or guided by experimental NMR data.

**Figure 1:**
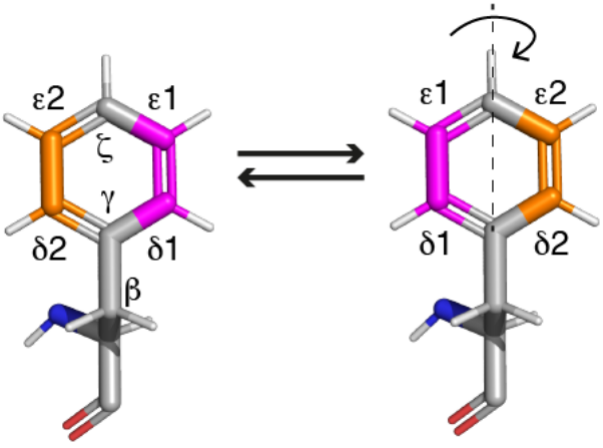
Schematic illustration of a phenylalanine residue undergoing ring flips, i.e., 180° rotations around the C^β^–C^γ^–C^ζ^ axis. The C^δ1^ (or C2) and C^ε1^ (C3) atoms are colored magenta, while C^δ2^ (C6) and C^ε2^ (C5) are colored orange. Because of symmetry, the two conformations are identical and consequently have equal populations, *p*_1_ = *p*_2_ = 0.5.

Aromatic side chains are prevalent in protein binding sites and perform functional roles in enzymatic catalysis, which motivates investigations of their dynamic properties. Moreover, aromatic residues form an integral part of the hydrophobic core of proteins, where they contribute roughly 25% of the volume on average. Despite the typically tight packing of aromatic rings in the protein core, Phe and Tyr aromatic rings undergo so-called ring flips, i.e., 180° rotamer transitions of the χ_2_ dihedral angle, alternatively described as 180° rotations around the C^β^–C^γ^–C^ζ^ axis (Figure 1). Aromatic ring flips are a hallmark of transient conformational fluctuations in proteins. The discovery of ring flips dates back to the mid-1970s, when it fundamentally changed the view of proteins by demonstrating their highly dynamic character.^9,10^ Rotation of an aromatic side chain packed inside the protein core requires that the surrounding residues transiently create sufficient void volume to accommodate the motion: The volume of the sphere swept out by a rotating ring is 164 Å^3^, whereas the volume of the ring itself is 125 Å^3^, indicating that ring flips require an activation volume of approximately 40 Å^3^. Naturally, one expects the activation volume to depend sensitively on the structural environment in each specific case. For example, proximal cavities are likely to reduce the activation volume, but it should also be recognized that the transient structural change might lead to an activation volume greater than the minimal requirement. Thus, ring flips serve as a proxy for complex protein dynamics that likely involve numerous side chains. By studying ring flips of Phe and Tyr residues in different types of environments, e.g., inside the protein core or on the surface, and as a function of physical parameters, e.g., temperature or pressure, it is possible to extract unique information about this type of dynamic process in terms of its dependence of molecular structure and its thermodynamic activation parameters. Early ground-breaking work by Wagner and Wüthrich revealed great variability in flip rate constants among aromatic rings located in different environments and also provided the first characterization of the activation barriers and activation volumes.^10−13^ However, despite the long history of studying ring flips, the number of proteins for which ring flip rate constants have been measured quantitatively is still very low.^11−24^ The scarcity of ring flip data is explained by the special challenges in characterizing ring flip rate constants quantitatively, as compared to measurements of many other types of conformational exchange. In addition, the typically slow time scale of ring flips makes them difficult to sample by MD simulations. Here we provide a perspective on how these challenges can be overcome with recent advances and offer an outlook on potential future developments. We focus primarily on selective isotope enrichment protocols and NMR relaxation dispersion experiments on proteins in solution but also highlight recent studies and potential future contributions of solid-state NMR and MD simulations.

## Studying Conformational Exchange by NMR—a Short Primer

**Figure 2:**
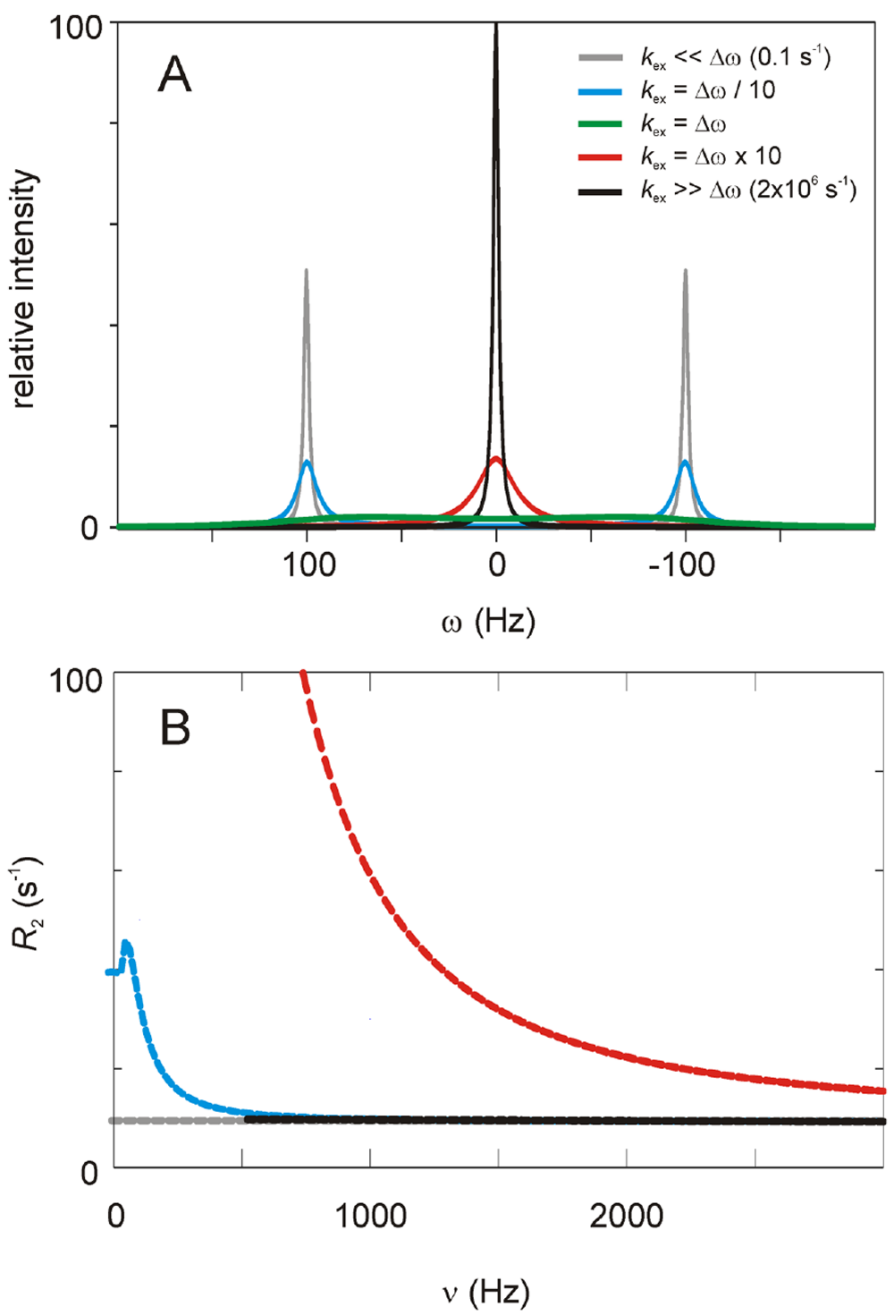
Effects of ring flips on the NMR spectrum and relaxation behavior of aromatic sites. (A) Effect of ring flips on the intensity, line width, and observed frequency of NMR signals. The resonance frequencies of the two symmetry-related positions on either side of the ring are ω/(2π) = ±100 Hz. The different exchange cases are *k*_ex_ ≪ Δω (0.1 s^–1^, gray), *k*_ex_ = Δω/10 (cyan), *k*_ex_ = Δω (green), *k*_ex_ = Δω × 10 (red), and *k*_ex_ ≫ Δω (2 × 10^6^ s^–1^, black). The relative populations of the two sites are equal due to symmetry. (B) Relaxation dispersion profiles corresponding to different exchange cases outlined in panel A. The black and gray lines overlap throughout the plotted region.

Exchange between alternative conformational states manifests in the NMR spectrum by affecting peak positions and line widths, provided that the interaction strength of the spin (with the local magnetic field or other spins) differs between the alternative conformations. The most common case is a difference in the local magnetic field experienced by the spin, arising from chemical shielding by the surrounding electrons of the nuclear spin from the static magnetic field. Whenever the chemical shift differs between two sides of an aromatic ring (Figure 1A), the ring flip rate constant will affect the appearance of the spectrum. In the slow exchange regime, *k*_ex_ < Δω, two separate peaks are observed for the two different positions on either side of the aromatic ring (Figure 2A, gray and cyan). The line widths of the two peaks depend on *k*_ex_ in relation to Δω. In the intermediate regime, the line width reaches its maximum for *k*_ex_ = Δω (Figure 2A, green). In the fast exchange regime, *k*_ex_ > Δω, a single peak is observed at the population-weighted chemical shift (Figure 2A, red and black), which in the case of aromatic ring flips appears at the midpoint between the two isolated peaks observed under slow exchange conditions because the populations are equal. Thus, the appearance of two separate peaks directly sets a limit on the exchange rate. By contrast, the observation of a single peak does not contain any information about the ring flip rate, unless the value of Δω is known. It is possible in some cases to slow down exchange by lowering the temperature or increasing pressure, so that separate peaks appear for the two sites.

In the fast-exchange limit (Figure 2B, red), the exchange contribution (*R*_ex_) to the line width is governed by the product of the two populations and the chemical shift difference (in units of radians per second) divided by the exchange rate constant, *p*_1_*p*_2_Δω/*k*_ex_. In the slow-exchange limit (Figure 2B, cyan), the lifetime broadening of the NMR signals imparts *k*_ex_/2 to the line widths. The exchange contribution to the line widths can be manipulated by the application of refocusing radio frequency (RF) fields implemented as a train of 180° pulses in CPMG experiments or a continuous RF field in *R*_1ρ_ experiments. By measuring the relaxation rate constant as a function of the refocusing frequency, ν_RF_, it is possible to determine the relaxation dispersion curve to which model parameters can be fitted (Figure 2B). General expressions have been derived to describe how *R*_ex_ depends on the underlying exchange parameters (*p*_*i*_, Δω_*ij*_, *k*_*i*_) and the experimental variable ν_RF_.^25,26^

Aromatic ring flips constitute a special case of exchange where the population factor is at a maximum (*p*_1_ = *p*_2_; *p*_1_*p*_2_ = 0.25). By contrast, in the case of exchange involving a weakly populated high-energy state, it is often the case that *p*_1_*p*_2_ ≈ 0.01. For aromatic sites, this feature is both a blessing and a curse because it reduces the number of fitting parameters and enhances the exchange effect, the latter of which may be beneficial, but it can also cause excessive line-broadening making measurements impossible unless Δω/*k*_ex_ is small. In the latter case, it might be possible to vary the experimental parameters, e.g., temperature or pressure, in order to find conditions that are more suitable for detailed characterization of ring flip processes.

As more studies on aromatic ring flips have been reported, the accumulated results indicate that Δω in many cases is relatively small, as might also be inferred from the relatively limited chemical shift dispersion observed for aromatic ^13^C spins in Phe residues, which have a standard deviation of approximately σ_δ_ = 1.2 ppm, compared to σ_δ_ = 2.0–3.4 ppm for ^13^C methyl shifts or σ_δ_ = 2.1–4.6 ppm for ^13^C^α^ shifts.^27^ This means that the fast-exchange condition can be met even though the actual flip rate might be fairly slow in absolute terms.

## Setting the Stage for Detailed Investigations of Ring Flips

As mentioned above, there are methodological challenges to studying ring flips. NMR studies of ring flips are hampered by the inherent properties of the spin systems in Phe and Tyr side chains, which puts special demands on the experimental design. Strong *J* coupling between vicinal carbons and also between vicinal protons makes it necessary to introduce site-selective isotope labels in order to avoid such interactions.^28−38^ In general, optimized labeling schemes for specific sites in aromatic side chains enable improved studies of ring flips and other types of dynamics. For example, by comparing relaxation rate constants measured for ^13^C^δ^ and ^13^C^ζ^ nuclei, it is possible to distinguish ring rotations from other types of exchange dynamics because the latter nucleus is not affected by rotations around the C^β^–C^γ^–C^ζ^ axis.^22,39^

Additional complications arise from the fact that aromatic rings located in the interior of proteins are often rigid on the subnanosecond time scale, resulting in relatively rapid relaxation. However, this problem can be counteracted by selecting the slowly relaxing multiplet component via so-called TROSY pulse schemes.^40,41^ Thus, methods development has resulted in an experimental toolbox that combines site selective ^13^C and/or ^2^H labeling with TROSY selection to enable a high-resolution view on the structure and dynamics of aromatic side chains. Of particular relevance to ring flip rate measurements, CPMG and *R*_1ρ_ relaxation dispersion experiments incorporating longitudinal relaxation optimized TROSY (L-TROSY) have been designed to enable accurate characterization of ring flip dynamics.^42,43^ To date, experiments performed on proteins in aqueous solution have enabled ring flip rate measurements for proteins below 15 kDa in molecular weight. It is expected that ring flip dynamics in quite large proteins should be possible to study using the L-TROSY approach,^42,43^ but this has not yet been fully tested. Recent development of ^19^F–^13^C TROSY methods potentially opens the window toward even larger systems and other time scales,^44,45^ although fluorine-substituted aromatic rings will likely have different interactions and dynamic properties than their native counterparts.

Solid-state NMR experiments offer complementary advantages to studying ring flips because dipolar couplings are averaged by molecular dynamics in such a way that the amplitude and anisotropy of the underlying motion can be determined.^23,24,39,46,47^ Furthermore, molecular size is not a limiting factor in solid-state NMR, as long as the analysis is not hampered by spectral overlap due to the increased number of aromatic spins. Potential drawbacks in solid-state NMR include the effects of the dipolar and chemical shift anisotropy relaxation mechanisms, which become very efficient if ring flips occur in the microsecond regime, thereby rendering the signals unobservable. The effect of crystal packing on ring flip dynamics in microcrystalline solid-state NMR samples is of potential concern.^24^ The detailed intermolecular interactions of exposed aromatic rings can play significant roles in governing ring flip rate constants,^24,47,48^ but it remains an open question to what extent crystal packing might influence the dynamics of buried rings; apparent discrepancies between solution-state and solid-state NMR data obtained for the small protein GB1 suggest that crystal packing might play a role also in this case.^46,49^ Further comparative studies are needed in this area.

## Computational Studies to Augment and Interpret NMR Data

Ring flips can be investigated computationally to augment experimental results from NMR, as was recognized in early investigations.^50^ MD simulations provide mechanistic details of the actual ring flip process.^51−53^ Because ring flips of buried aromatic residues usually are rare events, occurring on time scales of microseconds to milliseconds, various types of accelerated MD are usually needed to achieve sufficient sampling of the barrier crossing and to reach statistically stable results.^53,54^ Nonaccelerated MD simulations allow for qualitative characterization of ring flips into fast (observed frequently in an MD trajectory) or slow (not or rarely observed) categories.^24,51^ To date, MD simulations have not been able to reliably reproduce experimentally determined ring flip rate constants,^20,51^ indicating that NMR data provide valuable benchmarks and further highlighting the need for continued, data-guided development of force fields. MD simulations can also be directly guided by NMR data, either through reweighting of conformational ensembles or introducing restraints.^55−58^ We foresee that significant insights into the mechanisms of ring flips will be attained by combining MD simulations and NMR data describing ring flip dynamics and activation parameters as well as fast time scale fluctuations within the ground-state basin of the aromatic ring itself and surrounding residues.

## Ring Flip Activation Parameters from Temperature- and Pressure-Dependent Measurements

In addition to measuring ring flip rate constants, it is of great interest to determine the activation barriers as a means to characterize the nature of the collective “breathing” motions that enable ring flips. The activation barrier can be characterized in terms of thermodynamic activation parameters, including the enthalpy (Δ*H*^‡^), entropy (Δ*S*^‡^), and volume (Δ*V*^‡^).^11−17,20−22,59^ These parameters can be determined from temperature- and pressure-dependent ring flip rate measurements.

High-pressure NMR has become an established and increasingly widespread method,^60,61^ making it possible to study the pressure dependence of ring flips, covering a range from atmospheric pressure and up to several kilobar. A few reports have utilized a combination of temperature and pressure dependence to characterize all three activation parameters as well as additional parameters, such as the activation compressibility factor, which provide considerable additional insights into the underlying dynamics of the protein core.

**Figure 3:**
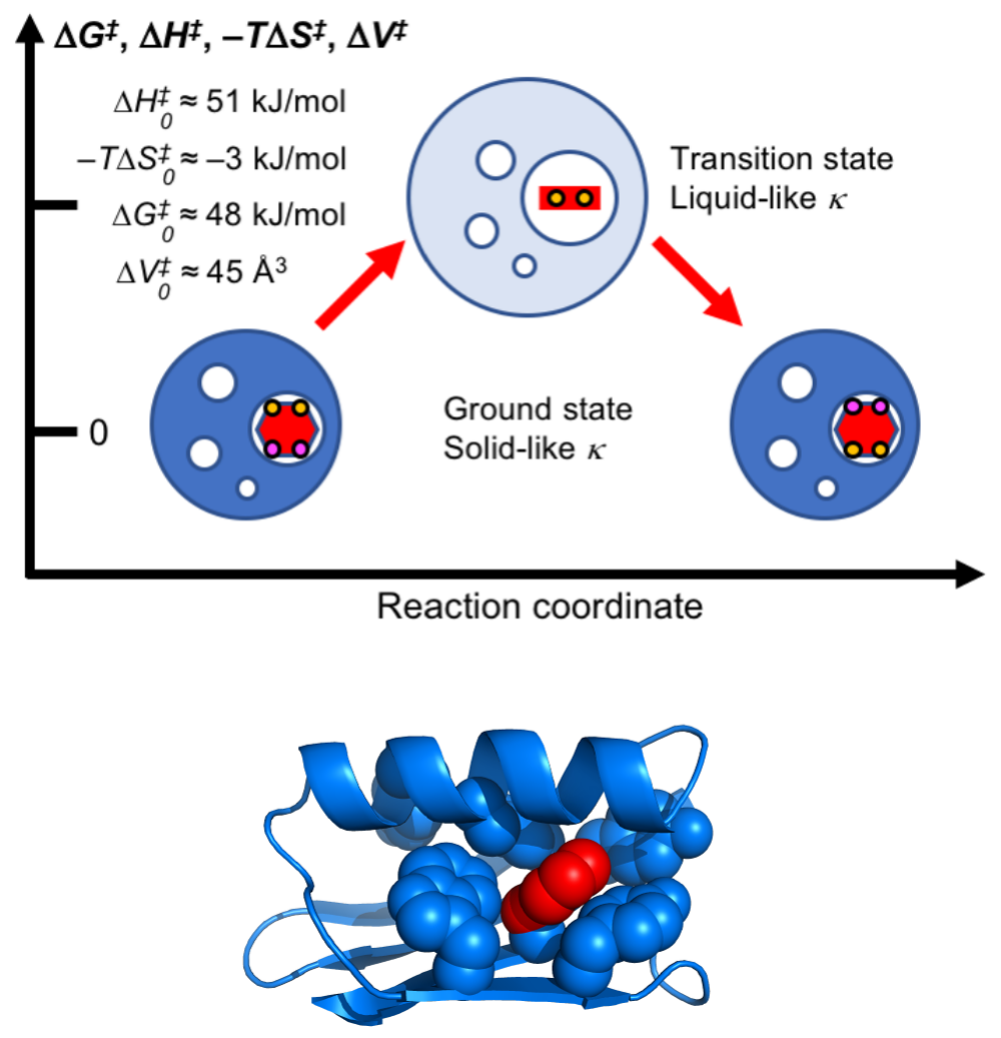
Schematic illustration of a ring flip process. The blue spheres represent the protein with void volumes shown as white spheres and the aromatic ring in red. The ground state is shown in dark blue with the aromatic ring as a red hexagon and the C^δ^ and C^ε^ atoms colored as in Figure 1. The transition state is shown in light blue with the aromatic ring as a red line indicating an orientation orthogonal to that in the ground state. The relative size of the spheres provides a qualitative indication of volume changes. Example thermodynamic data are given for F52 in protein GB1 at a standard state of *T* = 293 K and *p* = 1 bar. Figure adapted from ref (59). The bottom panel shows the location of F52 in the ground-state structure, where the aromatic ring is tightly packed in the protein interior with a solvent accessible surface area of only 5 Å^2^. The protein backbone trace is shown in ribbon representation with the aromatic side chains as space-filling spheres. The bottom panel was prepared using PyMOL.^63^

Figure 3 provides a schematic illustration with example results from our recent work on protein GB1.^59^ To date, temperature-dependent studies have determined Δ*H*^‡^ and Δ*S*^‡^ for a small number of proteins, whereas pressure-dependent studies to determine Δ*V*^‡^ have been applied in just five cases on three different proteins.^11,15,17,21,59^ It should be noted that the relatively few published reports are likely biased toward slower ring flip rate constants due to challenges in quantifying faster rate constants (as noted above); consequently, the available data on barriers are presumably similarly biased toward high barriers. The Δ*H*^‡^ values determined to date range from 50 to 150 kJ mol^–1^, while Δ*S*^‡^ varies between 5 and 300 J mol^–1^ K^–1^ and Δ*V*^‡^ between 45 and 84 Å^3^. This variability reflects the differences in packing interactions of the aromatic ring and the extent of structural changes required to create the transition-state volume enabling the ring to flip.

We recently determined the change in compressibility (Δκ^‡^) between the ground and transition states by performing a combined analysis of temperature- and pressure-dependent flip rate constants for the buried ring of F52 in protein GB1.^59^ Our results indicate that the transition state of the ring flip is liquid-like with a compressibility similar to that of short-chain alkanes, whereas the ground state is solid-like in agreement with previous studies of protein compressibility (Figure 3). We believe that the high compressibility of the transition state reflects significant loss of structural order in the neighborhood of the ring. Furthermore, by extrapolating our results to higher pressures, on the basis of the derived model, we arrived at the conclusion that ring flips can occur even when Δ*V*^‡^ ≈ 0. In this case, the transient volume expansion required in the immediate surroundings of the ring appears to be compensated by compaction of remote void volumes, such that there is no net expansion of the protein core. This notion further suggests that ring flips might be accommodated by cavity migration through the protein core, a phenomenon that has been observed in MD simulations.^62^ The unique information about the transition state obtained by performing pressure- and temperature-dependent studies motivates further work along these lines. Future work will likely provide additional insights into the relationship between activation parameters and interactions of the aromatic ring with the surrounding residues in the ground- and transition-state ensembles.

## Ring Flip Mechanisms

Little is currently known about the detailed mechanisms of ring flips inside the core of globular proteins. While it is clear that ring flips require that neighboring atoms move away to create sufficient space for the ring to rotate, the details of such a process are not clear. Naturally, one would expect a large variety of scenarios among different proteins depending on the structural environment of the flipping ring.

Ideally, we would like to obtain high-resolution structural view of the entire ring flip process, but experimental data are heavily dominated by dynamics and energetics, i.e., rate constants and activation barriers that need to be interpreted in structural terms. Analysis of MD simulations can yield this type of detailed information, which can be further validated to some extent by experimental data, as described above. Below we highlight recent cases that have provided insights into the mechanisms of ring flips.

**Figure 4:**
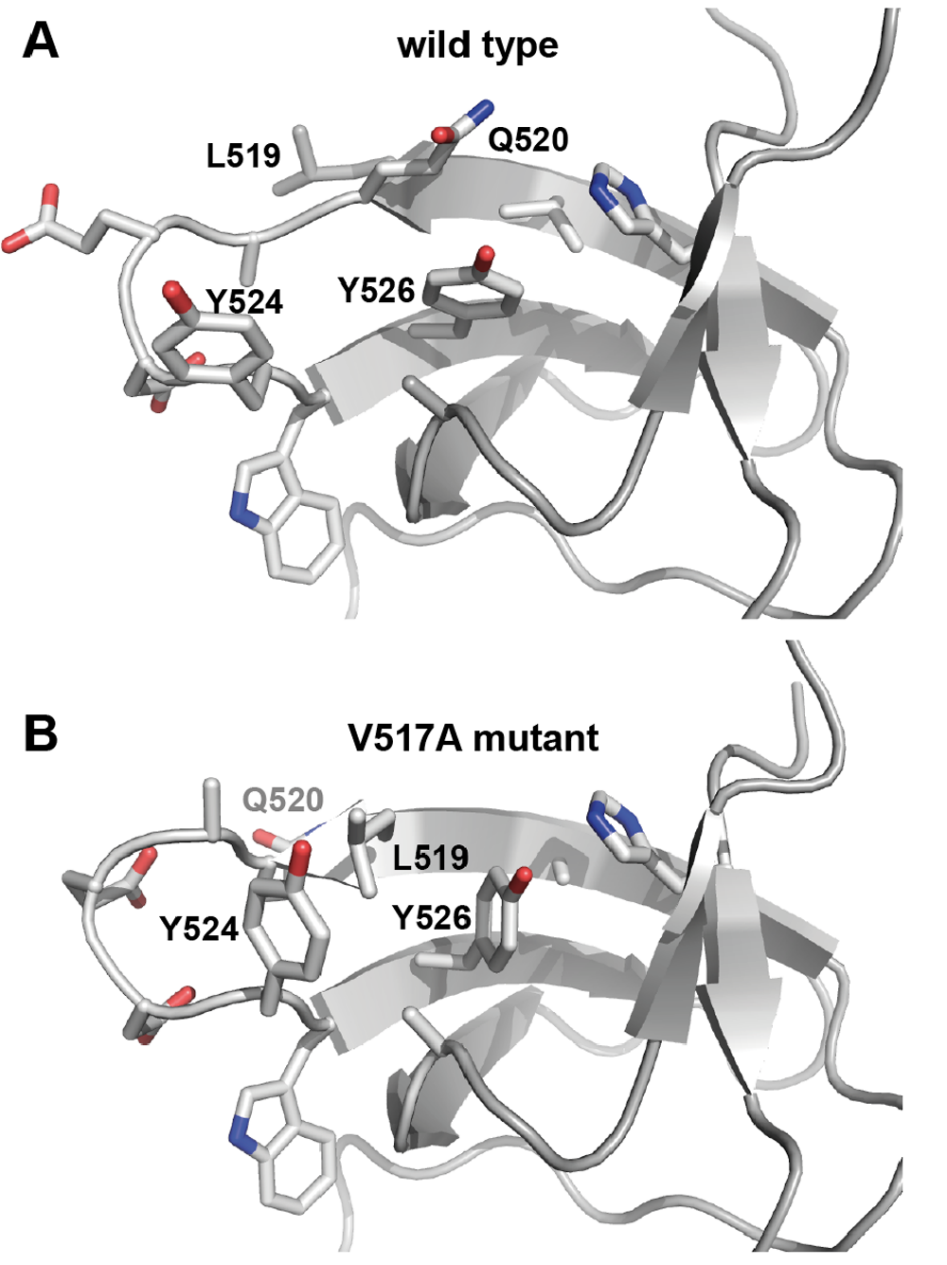
Model of conformational changes enabling ring flips. Comparison of (A) wild-type (PDB id 2FPE(64)) and (B) mutant (PDB id 7NYM(52)) variants of an SH3 domain shows a change in the χ_2_ dihedral angle of Y526 from eclipsed to staggered that is accompanied by a local inversion of the β strand at residues 518–520.^52^ The wild-type structure represents the ground-state conformation, while the mutant structure represents a high-energy state that is off pathway with respect to the ring-flip reaction coordinate. The figure was prepared using PyMOL.^63^

Using a combination of mutational analysis, X-ray crystallography, NMR relaxation dispersion, and microsecond long MD simulations, Ringkjøbing Jensen and co-workers studied a tyrosine residue (Y526) in an SH3 domain.^52^ In addition to undergoing full ring flips, Y526 exchanges between major (97%) and minor (3%) conformations that differ in the χ_2_ dihedral angle. The high-resolution minor state structure, stabilized by mutations and solved by X-ray crystallography, reveals large-scale rearrangements of neighboring side chains and inversion of a β strand to accommodate the change in the χ_2_ dihedral angle of Y526 (Figure 4). MD simulations further showed how a structural change of the neighboring side chains creates an expansion by 65 Å^3^ of void volume around the Y526 ring, which enables ring flipping. However, the rate constant for the major to minor transition was determined to 70 s^–1^, whereas the rate constant for Y526 ring flips was estimated to *k*_flip_ > 25000 s^–1^. Thus, the ring flip occurs much more frequently than the major–minor transition, indicating that the minor conformation need not be closely representative of any conformation along the ring-flip trajectory; rather, the minor conformation involves additional stabilizing interactions between the Y526 ring and the surrounding residues that are not present during a productive ring flip trajectory, but occasionally trap the ring in the minor conformation. Nevertheless, the structural difference between the major and minor conformations do provide important insights into the types of large-scale breathing motions of the protein core that might occur during a ring flip.

**Figure 5:**
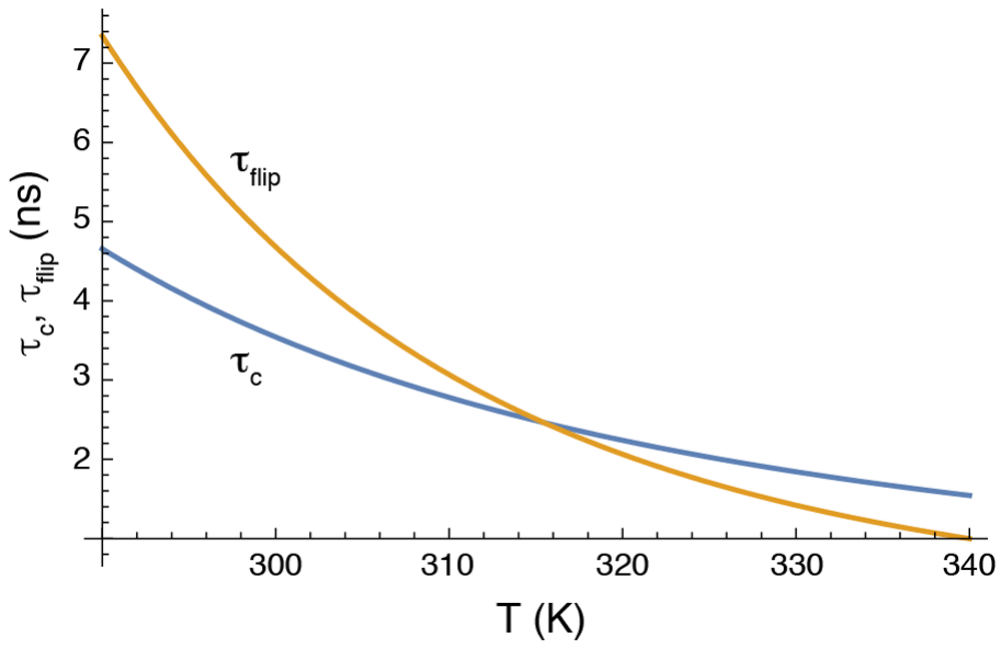
Temperature dependence of correlation times for overall rotational diffusion and ring flips. The correlation time for overall rotational diffusion, τ_c_, was calculated using the Stokes–Einstein equation: τ_c_ = *V*_H_η/(*k*_B_*T*), where *V*_H_ is the hydrodynamic volume of the protein, η is the viscosity of water, calculated using the Vogel–Fulcher–Tammann equation as η = 2.939 × 10^–5^ exp[507.88/(*T* – 149.3)] Pa s, and *k*_B_ is Boltzmann’s constant. The correlation time for ring flips, τ_flip_, was calculated as 1/*k*_flip_, with *k*_flip_ = (*k*_B_*T*/*h*) exp[−Δ*G*^‡^ + Δ*S*^‡^(*T* – 310)/(*N*_Av_*k*_B_*T*)], where *h* is Planck’s constant, Δ*G*^‡^ = 25.5 kJ/mol is the activation free energy at the reference temperature, Δ*S*^‡^= 15 J/(K mol) is the activation entropy, and *N*_Av_ is Avogadro’s number. Other parameters might yield steeper or less steep dependence of τ_flip_ on *T*.

A very different example is provided by Wand and co-workers, who studied the dynamics of all three aromatic residues (F4, F45, and Y59) in ubiquitin,^65^ all of which have a relatively high degree of solvent accessibility. The Wand team determined the temperature dependence of fast-time scale order parameters, which represent the amplitude of motions on time scales shorter than the overall rotational correlation time (τ_c_). The results showed an abrupt change in order parameter from high (0.75–0.95) to low (0.35–0.55) over a 20° increase in temperature from 305 to 325 K, which the authors interpreted as the onset of a qualitatively different type of motion. They suggested that ring motion is largely librational below the transition temperature, whereas complete ring rotation sets in above the transition temperature and then occurs as continuous rotational diffusion. However, we propose an alternative interpretation of the data that is consistent with the traditional concept of jump-like barrier crossing throughout the studied temperature range. In order to affect the order parameter, a ring flip must occur with a correlation time shorter than τ_c_, i.e., τ_flip_ = 1/*k*_flip_ < τ_c_, which is on the order of nanoseconds. We believe that the abrupt change in order parameter arises, at least in part, because the correlation time of ring flips might decrease more rapidly with increasing temperature than does τ_c_. To illustrate this concept, Figure 5 shows the temperature dependence of τ_c_ and τ_flip_, where the latter value is calculated based on a plausible free-energy barrier. Our model suggests that there need not exist any abrupt, qualitative change in motion of the aromatic ring; instead, the ring is undergoing rapid ring flips throughout the temperature range. Notably, the sharp transition of the order parameters thus enables an estimate of the ring flip rate constant, which is equal to roughly 1/τ_c_ at the transition temperature and similar to results obtained for solvent exposed aromatic rings in peptides.^66,67^ Recent solid-state NMR studies of phenylalanine dynamics in ubiquitin crystals support this interpretation for F4, with respect to both the nanosecond time scale and jump-like character of the ring flips.^44^ However, that study also noted that F45 is exchange broadened beyond detection in the crystalline state, suggesting that it is undergoing exchange on the microsecond time scale. While the NMR data do not establish that the exchange is due to ring flips, MD simulations support the interpretation that F45 undergoes ring flips with a correlation time on the order of 1 μs, rather than nanoseconds.^24^ The results on ubiquitin provide a nice example of very fast ring flips of aromatic side chains located close to the protein surface, showing that the barriers of rotation in these cases can approach that derived from the χ_2_ dihedral angle potential function, but also be significantly higher.

Very recently, we measured ring flip rate constants using ^13^C *R*_1ρ_ relaxation dispersion caused by residual dipolar coupling (RDC) mediated exchange broadening in weakly aligned proteins.^49^ This approach, first demonstrated by the Kay and Palmer groups for ^15^N backbone amides and ^13^C methyl groups, respectively,^68,69^ enables measurement of exchange rates even when there is no or very small chemical shift difference between the exchanging sites—which appears to be a relatively common scenario in aromatic side chains, as already mentioned above. The RDC involving the covalently bonded ^1^H–^13^C nuclei depends on the orientation of the bond vector with respect to the static magnetic field axis and thus carries powerful structural information. Ring flipping amounts to a reorientation by 120° of the exchanging sites, which in most cases leads to a significant change in RDC, unless the bond vectors in the two symmetry-related sites are oriented identically with respect to the static magnetic field axis. The difference in RDC between the two exchanging sites, Δω_RDC_, can be compared with that calculated from various models of the exchange mechanism, including the continuous diffusion model suggested previously.^65^ Thus, we compared the experimentally determined Δω_RDC_ with the value determined from the static structure, i.e., the X-ray crystal structure, as well as the ensemble-averaged value expected for a continuous rotational diffusive motion around the χ_2_ dihedral angle, assuming a distribution corresponding to the CHARMM36 potential function for χ_2_, which is the simplest realistic energy landscape in which rotational diffusion of the aromatic ring can occur. The experimental Δω_RDC_ determined for F52 in GB1 fits perfectly with the value calculated from the X-ray crystal structure. By contrast, the models describing continuous diffusive rotation show very poor agreement with the experimental Δω_RDC_. Thus, the ring of F52 in GB1 spends much longer time exploring the free energy basin of each rotamer than it does traversing the energy barrier of the χ_2_ rotamer change, indicating that steric restraints from the surrounding protein residues restrain the fluctuation amplitudes within each basin. The results directly demonstrate that the ring flip occurs as a rare jump-like transition in this case of a buried phenylalanine residue inside a small, globular protein. Not surprisingly, the different environments of this aromatic ring and the more highly surface-exposed aromatic residues in ubiquitin give rise to quite different ring flip dynamics.

The three studies described above, which in various ways provide insights into ring flip mechanisms, have all relied on relatively recent methodological developments. We expect that future studies on different systems, potentially using new approaches, will lead to an increasingly detailed description of ring flip mechanisms and their dependence on the structure, dynamics, and energetics of the accommodating environment.

## Outlook and Concluding Remarks

Today, we have available the methods needed to investigate aromatic ring flip dynamics in proteins over a range of time scales. As we have outlined above, detailed information about ring flip dynamics in terms of rate constants, activation parameters, and, to some extent, mechanisms can be attained using NMR relaxation experiments conducted in a temperature- and/or pressure-dependent manner. Future studies employing this methodology will continue to address fundamental questions of protein dynamics. How do ring flip rate constants depend on protein size, packing density, and the extent of solvent exposure? How do structural features like clusters of aromatic side chains affect ring flips? Are the ring flips of residues forming such clusters correlated? How do specific interactions like hydrogen bonding (in the case of tyrosine), π–π, cation−π, or CH−π interactions affect ring flip dynamics? More broadly, it is of great interest to establish a database correlating experimentally measured ring flip rate constants with protein structural features. Through these types of studies, we aim to obtain insights into the physicochemical nature of the hydrophobic core.

Determining the activation volume and compressibility difference between the ground state and the transition state of the ring flip for an extended set of aromatic residues will allow us to attain novel insights into the physical properties of the surrounding protein “matrix”. How solid-like or liquid-like are different regions of proteins? Our understanding of these issues will likely benefit from studying larger proteins that present opportunities to probe ring flip dynamics of residues located at different depths from the protein surface.

Another obvious avenue for future work is to address the relationship between the amplitudes and time scales of fast time scale fluctuations within the rotamer basins and compare these with the slower time scales and energetics of ring flip barrier crossings. Fast time scale fluctuations in proteins can be routinely investigated by NMR and MD simulations, but previous NMR studies addressing this time scale have primarily focused on backbone amides and side-chain methyl groups and have so far produced only a few reports on aromatic side chains.^32,65,70,71^ Future studies combining MD simulations and NMR applied to the same protein will be able to address both fast time-scale fluctuations and ring flip dynamics to reveal the possible connection between these dynamic processes of different amplitudes and different time-scales. The combination of solution-state and solid-state NMR could be particularly powerful in this regard.

To conclude, the stage is set for future in-depth studies of aromatic ring flips to probe fundamental dynamic properties of proteins involving multiple residues. We anticipate that many insightful reports will appear over the coming years to unveil the molecular details underlying the great variability of ring flips rate constants and the complex nature of the coupled dynamics involving the surrounding residues.

## Abbreviations

CPMG: Carr–Purcell–Meiboom–Gill
MD: molecular dynamics
GB1: streptococcal protein G domain B1
RDC: residual dipolar coupling
TROSY: transverse relaxation optimized spectroscopy.
